# Supplementary material for: Revisiting paramagnetic relaxation enhancements in slowly rotating systems: how long is the long range?
Source: Magn Reson (Gott). 2021 Jan 29;2(1):25–31. doi: 10.5194/mr-2-25-2021 (PMC10539754; doi:10.5194/mr-2-25-2021)
Supplement: The supplement related to this article is available online at: https://doi.org/10.5194/mr-2-25-2021-supplement. [file mr-2-25-supplement.pdf]

Supplement of Magn. Reson., 2, 25–31, 2021
https://doi.org/10.5194/mr-2-25-2021-supplement
© Author(s) 2021. This work is distributed under
the Creative Commons Attribution 4.0 License.

Open Access
MAGNETIC RESONANCE

Supplement of

Revisiting paramagnetic relaxation enhancements in slowly rotating systems: how long is the long range?

Giovanni Bellomo et al.

Correspondence to: Claudio Luchinat (luchinat@cerm.unifi.it)

The copyright of individual parts of the supplement might differ from the CC BY 4.0 License.

The complete relaxation rate-matrix approach

The time dependence of the longitudinal magnetization due to the dipole-dipole interaction between two magnetically unlike spins *I* and *J* of equal spin quantum number (*I* = *J*) is described by (Bertini et al., 2017; Solomon, 1955)

$$\frac{\mathrm{d}M_z^I}{\mathrm{d}t} = -\rho^I \left(M_z^I - M_{eq}^I\right) - \sigma^{IJ}\left(M_z^J - M_{eq}^J\right) \tag{S1a}$$

$$\frac{\mathrm{d}M_z^J}{\mathrm{d}t} = -\rho^J \left(M_z^J - M_{eq}^J\right) - \sigma^{JI}\left(M_z^I - M_{eq}^I\right) \tag{S1b}$$

where M_{eq} is the equilibrium magnetization, with

$$\rho^I = w_0 + 2w_1^I + w_2 \tag{S2a}$$

$$\rho^J = w_0 + 2w_1^J + w_2 \tag{S2b}$$

$$\sigma^{IJ} = \sigma^{JI} = w_2 - w_0 \, . \tag{S3}$$

The terms w_0, w_1 and w_2 indicate the zero, single and double quantum spin transition probabilities, respectively: w_0 is the probability of transition simultaneously causing a decrease in m_I and an increase in m_J, or vice versa (zero quantum transitions), and is equal to

$$w_0 = \frac{2}{15}\left(\frac{\mu_0}{4\pi}\frac{\hbar^2\gamma_I\gamma_J}{r_{IJ}^3}\right)^2 J(J+1)\frac{\tau_c}{1+\left(\omega_I-\omega_J\right)^2\tau_c^2} \tag{S4}$$

where r_{IJ} is the internuclear distance; w_1^I is the probability of single transitions between states with the same m_J and different m_I (single quantum transitions)

$$w_1^I = \frac{1}{5}\left(\frac{\mu_0}{4\pi}\frac{\hbar^2\gamma_I\gamma_J}{r_{IJ}^3}\right)^2 J(J+1)\frac{\tau_c}{1+\omega_I^2\tau_c^2} \tag{S5}$$

and w_2 indicates the probabilities of transitions causing a decrease, or an increase, in both m_I and m_J (double quantum transitions)

$$w_2 = \frac{4}{5}\left(\frac{\mu_0}{4\pi}\frac{\hbar^2\gamma_I\gamma_J}{r_{IJ}^3}\right)^2 J(J+1)\frac{\tau_c}{1+\left(\omega_I+\omega_J\right)^2\tau_c^2} \tag{S6}$$

In the assumption of a completely rigid spherical molecule, the correlation time τ_c is the isotropic molecular reorientation time.

The cross relaxation rates $\sigma^{IJ} = \sigma^{JI}$ describe the effect on the variation of the magnetization of one spin due to the variation of the magnetization of the other spin, resulting from their interaction.

By definition of a magnetization vector $\mathbf{M} = \begin{pmatrix} M_z^I \\ M_z^J \end{pmatrix}$ and of a relaxation matrix $\mathbf{R} = \begin{pmatrix} \rho^I & \sigma^{IJ} \\ \sigma^{JI} & \rho^J \end{pmatrix}$, Eqs. 1 can be written in the matrix form

$$\frac{\mathrm{d}\mathbf{M}}{\mathrm{d}t} = -\mathbf{R}\cdot\left(\mathbf{M} - \mathbf{M_{eq}}\right) \tag{S7}$$

so that

$$\mathbf{M}(t) - \mathbf{M_{eq}} = \exp(-\mathbf{R}t)\cdot(\mathbf{M}(0) - \mathbf{M_{eq}}) \tag{S8}$$

In the presence of a rigid macromolecule with N atoms of the ^{1}H nuclide, the relaxation matrix becomes

$$\mathbf{R} = \begin{pmatrix} \rho_1 & \sigma_{12} & \sigma_{13} & \cdots & \sigma_{1N} \\ \sigma_{12} & \rho_2 & \sigma_{23} & \cdots & \sigma_{2N} \\ \sigma_{13} & \sigma_{23} & \rho_3 & \cdots & \sigma_{3N} \\ \vdots & \vdots & \vdots & \cdots & \vdots \\ \sigma_{1N} & \sigma_{2N} & \sigma_{3N} & \cdots & \rho_N \end{pmatrix} \tag{S9}$$

where

$$\rho_i = \sum_{j=1, j\neq i}^{N} \rho_{ij} \tag{S10}$$

with (see Eqs. 2)

$$\rho_{ij} = \frac{2}{15}\left(\frac{\mu_0}{4\pi}\frac{\hbar\gamma_I^2}{r_{ij}^3}\right)^2 I(I+1)\left[\tau_c + \frac{3\tau_c}{1+\omega_I^2\tau_c^2} + \frac{6\tau_c}{1+4\omega_I^2\tau_c^2}\right] \quad \text{(S11)}$$

if the *i*th and *j*th spin systems are magnetically non-equivalent (unlike spins), or by

$$\rho_{ij} = \frac{2}{5}\left(\frac{\mu_0}{4\pi}\frac{\hbar\gamma_I^2}{r_{ij}^3}\right)^2 I(I+1)\left[\frac{\tau_c}{1+\omega_I^2\tau_c^2} + \frac{4\tau_c}{1+4\omega_I^2\tau_c^2}\right] \quad \text{(S12)}$$

(corresponding to $\rho^I + \sigma^{IJ}$, see Eqs. 2a and 3), if *i*th and *j*th spin systems are magnetically equivalent (like spins), as, e.g., the methyl protons. The off-diagonal elements are

$$\sigma_{ij} = \frac{2}{15}\left(\frac{\mu_0}{4\pi}\frac{\hbar\gamma_I^2}{r_{ij}^3}\right)^2 I(I+1)\left[\frac{6\tau_c}{1+4\omega_I^2\tau_c^2} - \tau_c\right] \quad \text{(S13)}$$

Since **R** is a symmetric matrix, it can be diagonalized and written in the form

$$\mathbf{R} = \boldsymbol{\chi}\cdot\boldsymbol{\lambda}\cdot\boldsymbol{\chi}^{-1} \quad \text{(S14)}$$

where $\boldsymbol{\lambda}$ is the diagonal matrix of eigenvalues and $\boldsymbol{\chi}$ is the unitary eigenvector matrix. The program CORMA (Borgias et al., 1989) can be used to perform these calculations by providing the macromolecular structure in input.

From Eqs. 8 and 14, the time dependence of the *z*-component of the magnetization of each nuclear spin of the macromolecule can be calculated from the expression

$$\mathbf{M}(t) - \mathbf{M}_{\mathbf{eq}} = \boldsymbol{\chi}\cdot\exp(-\boldsymbol{\lambda}t)\cdot\boldsymbol{\chi}^{-1}\cdot(\mathbf{M}(0) - \mathbf{M}_{\mathbf{eq}}) \quad \text{(S15)}$$

where

$$\exp(-\boldsymbol{\lambda}t) = \begin{pmatrix} \exp(-\lambda_1 t) & 0 & 0 & \dots & 0 \\ 0 & \exp(-\lambda_2 t) & 0 & \dots & 0 \\ 0 & 0 & \exp(-\lambda_3 t) & \cdots & 0 \\ \vdots & \vdots & \vdots & \cdots & \vdots \\ 0 & 0 & 0 & \cdots & \exp(-\lambda_N t) \end{pmatrix}$$

with λ_i being the eigenvalues of the matrix **R**.

In the presence of chemical exchange, in the limiting case of a single nucleus in chemical exchange with bulk solvent nuclei,

$$I_1 \underset{k_{-1}}{\overset{k_1}{\rightleftharpoons}} I_B$$

the time evolution of the longitudinal magnetization is given by

$$\frac{d}{\mathrm{d}t}\begin{pmatrix} M_z^I \\ M_z^B \end{pmatrix} = -\begin{pmatrix} \rho_1^I + k_1 & -k_{-1} \\ -k_1 & \rho_B + k_{-1} \end{pmatrix}\begin{pmatrix} M_z^I - M_{eq}^I \\ M_z^B - M_{eq}^B \end{pmatrix}$$

and since at equilibrium $k_1 P_1 = k_{-1} P_B$ (P_1 is the population of the nuclei in position 1 and in the bulk), it results that $\frac{k_{-1}}{k_1} = \frac{P_1}{P_B} = f$.

The relaxation rates of the solvent molecule nuclei interacting with the macromolecule and of the bulk solvent molecule nuclei can be calculated by including in the relaxation matrix as many extra rows and columns as the number of nuclei belonging to the interacting solvent molecules, and an additional row and column relative to bulk solvent nuclei. Assuming M solvent nuclei interacting with the macromolecule (composed of N nuclei), the relaxation matrix becomes

$$\mathbf{R} = \begin{pmatrix} \rho_1 + k_1 + R_{1M,1} & \sigma_{12} & \cdots & \sigma_{1N} & \sigma_{1(N+1)} & \cdots & \sigma_{1(N+M)} & -fk_1 \\ \sigma_{12} & \rho_2 + k_2 + R_{1M,2} & \cdots & \sigma_{2N} & \sigma_{2(N+1)} & \cdots & \sigma_{2(N+M)} & -fk_2 \\ \vdots & \vdots & \ddots & \vdots & \vdots & \ddots & \vdots & \vdots \\ \sigma_{1N} & \sigma_{2N} & \cdots & \rho_N + k_N + R_{1M,N} & \sigma_{N(N+1)} & \cdots & \sigma_{N(N+M)} & -fk_N \\ \sigma_{1(N+1)} & \sigma_{2(N+1)} & \cdots & \sigma_{N(N+1)} & \rho_{N+1} + k_{N+1} + R_{1M,N+1} & \cdots & \sigma_{(N+1)(N+M)} & -fk_{N+1} \\ \vdots & \vdots & \ddots & \vdots & \vdots & \ddots & \vdots & \vdots \\ \sigma_{1(N+M)} & \sigma_{2(N+M)} & \cdots & \sigma_{N(N+M)} & \sigma_{(N+1)(N+M)} & \cdots & \rho_{N+M} + k_{N+M} + R_{1M,N+M} & -fk_{N+M} \\ -k_1 & -k_2 & & -k_N & -k_{N+1} & & -k_{N+M} & \rho_B + f\sum_i k_i \end{pmatrix} \quad \text{(S16)}$$

where $k_i = \left(\tau_{M,i}\right)^{-1}$ are the exchange rate constants, f is the ratio between the macromolecular concentration and the solvent molecule nuclei concentration, and ρ_B is the relaxation rate of bulk solvent nuclei in the absence of the macromolecule. The coefficient f in the last column originates from the relationship $k_{-i} = fk_i$ (see above).

Finally, using a "normalized" magnetization for the bulk nuclei, $\mathbf{M}' = \begin{pmatrix} M_z^I \\ \vdots \\ fM_z^B \end{pmatrix} = \begin{pmatrix} M_z^I \\ \vdots \\ \widetilde{M}_z^B \end{pmatrix}$, the relaxation matrix becomes

$$\mathbf{R}' = \begin{pmatrix} \rho_1 + k_1 + R_{1M,1} & \sigma_{12} & \cdots & \sigma_{1N} & \sigma_{1(N+1)} & \cdots & \sigma_{1(N+M)} & -k_1 \\ \sigma_{12} & \rho_2 + k_2 + R_{1M,2} & \cdots & \sigma_{2N} & \sigma_{2(N+1)} & \cdots & \sigma_{2(N+M)} & -k_2 \\ \vdots & \vdots & \ddots & \vdots & \vdots & \ddots & \vdots & \vdots \\ \sigma_{1N} & \sigma_{2N} & \cdots & \rho_N + k_N + R_{1M,N} & \sigma_{N(N+1)} & \cdots & \sigma_{N(N+M)} & -k_N \\ \sigma_{1(N+1)} & \sigma_{2(N+1)} & \cdots & \sigma_{N(N+1)} & \rho_{N+1} + k_{N+1} + R_{1M,N+1} & \cdots & \sigma_{(N+1)(N+M)} & -k_{N+1} \\ \vdots & \vdots & \ddots & \vdots & \vdots & \ddots & \vdots & \vdots \\ \sigma_{1(N+M)} & \sigma_{2(N+M)} & \cdots & \sigma_{N(N+M)} & \sigma_{(N+1)(N+M)} & \cdots & \rho_{N+M} + k_{N+M} + R_{1M,N+M} & -k_{N+M} \\ -fk_1 & -fk_2 & \cdots & -fk_N & -fk_{N+1} & \cdots & -fk_{N+M} & \rho_B + f\sum_i k_i \end{pmatrix} \quad \text{(S17)}$$

Bertini, I., Luchinat, C., Parigi, G. and Ravera, E.: NMR of paramagnetic molecules: applications to metallobiomolecules and models., 2017.

Borgias, B., Thomas, P. D. and James, T. L.: COmplete Relaxation Matrix Analysis (CORMA)., University of California, San Francisco, CA., 1989.

Solomon, I.: Relaxation Processes in a System of Two Spins, Phys. Rev., 99(2), 559–565, doi:10.1103/PhysRev.99.559, 1955.

Figure S1 shows the results of calculations for a system of six protons placed along a straight line at 10, 12, 14, 16, 18 and 20 Å from a gadolinium ion. The figure also shows (black dotted lines) the magnetization curves expected from the Solomon equation for the two protons at 10 and 20 Å. Clearly only the first points of the magnetization recovery for the proton at 10 Å agree with the relaxation rate predicted by the Solomon equation. In fact, the magnetization of the proton at 10 Å recovers its equilibrium value slower than predicted from an exponential behavior, so that the monoexponential fit provides a longer relaxation time. On the contrary, the magnetization recovery of the protons at the largest distances is steeper than predicted from an exponential behavior and the relaxation rates are sizably larger than predicted from the Solomon equation.

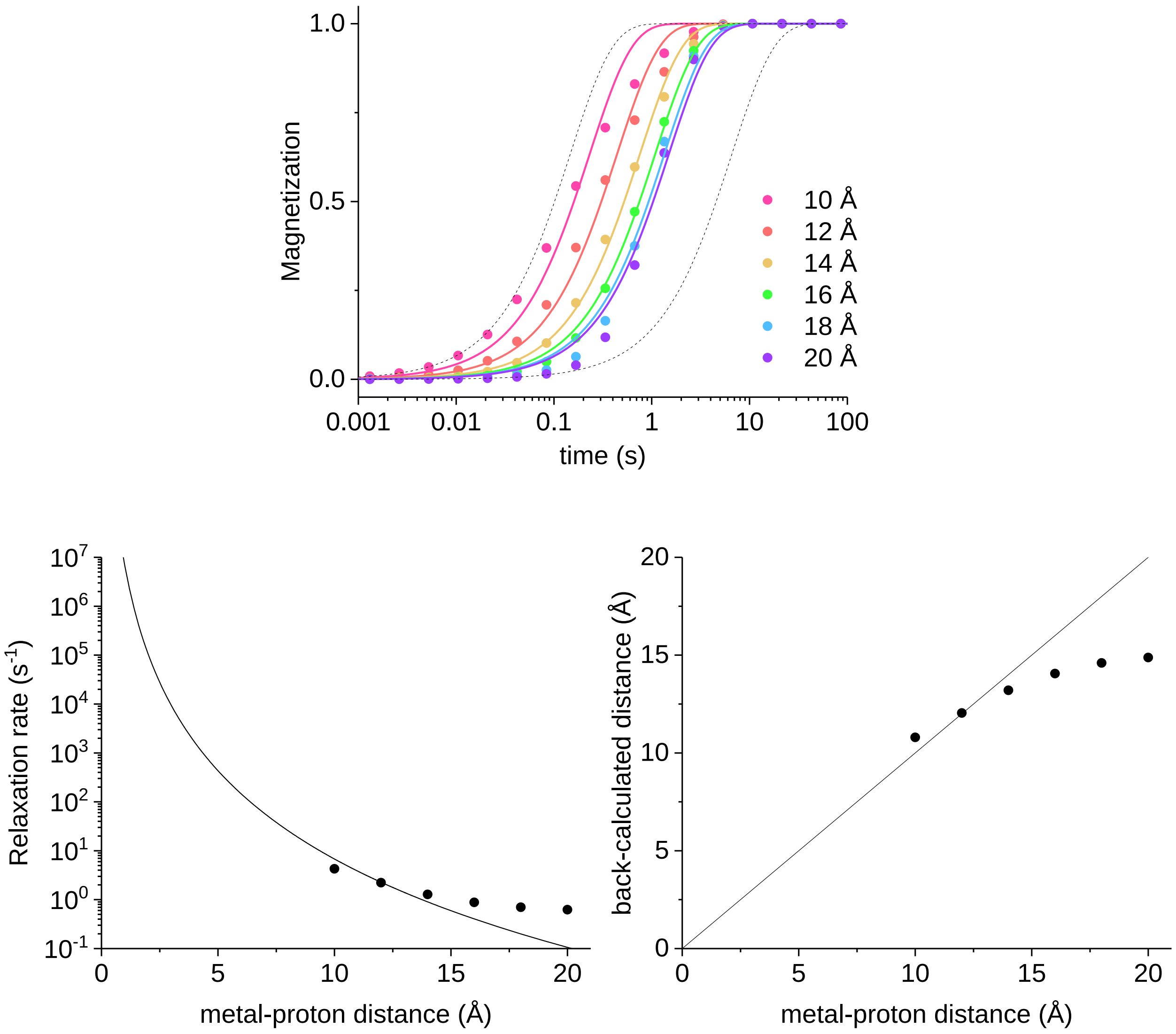

Figure S1. Calculated magnetization recovery for six protons placed along a straight line at 10, 12, 14, 16, 18 and 20 Å from a gadolinium ion, at 700 MHz (upper panel). The black dotted lines show the (monoexponential) behavior predicted from the Solomon equation for the two protons at 10 and 20 Å. The magnetization data calculated for the 6 protons are clearly not monoexponential. The monoexponential fits (solid colored lines) provide the relaxation rates and the back-calculated distances shown in the lower panels.

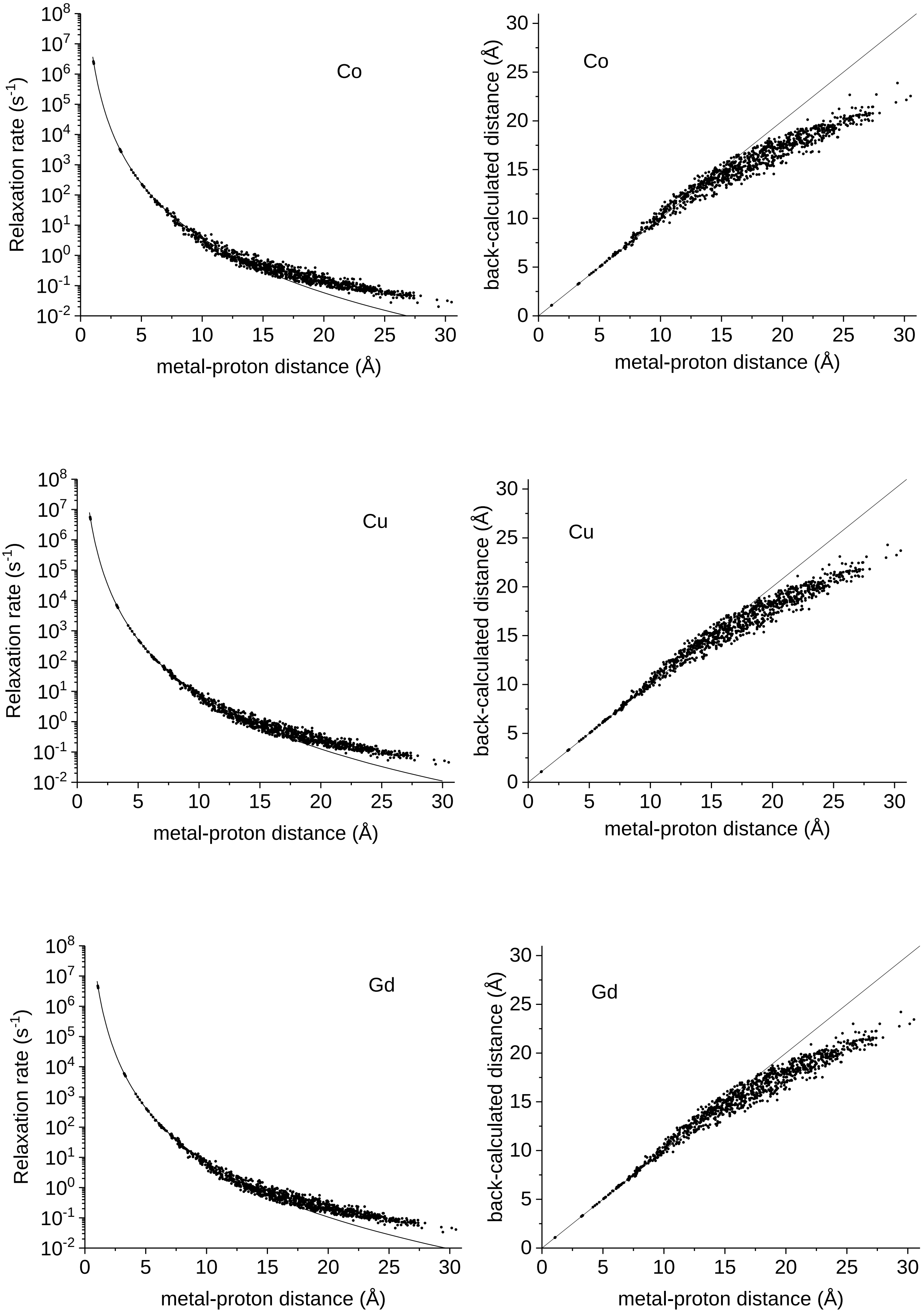
Relaxation rate (s-1)
Co
metal-proton distance (Å)
back-calculated distance (Å)
Co
metal-proton distance (Å)
Relaxation rate (s-1)
Cu
metal-proton distance (Å)
back-calculated distance (Å)
Cu
metal-proton distance (Å)
Relaxation rate (s-1)
Gd
metal-proton distance (Å)
back-calculated distance (Å)
Gd
metal-proton distance (Å)

Figure S2. (left panels) Paramagnetic relaxation rates calculated at 700 MHz for MMP-12 protons, with a reorientation time of 12 ns, in the presence of high spin cobalt(II) (with an electron relaxation rate of 10 ps), copper(II) (with an electron relaxation rate of 0.17 ns), or gadolinium(III) ions (with an electron relaxation rate of 1 μs), replaced to the catalytic zinc(II) ion. The lines indicate the rates predicted with the Solomon equation. (right panels) Agreement between metal-proton distances as measured in the PDB 5LAB structure and back-calculated from the predicted R_1.

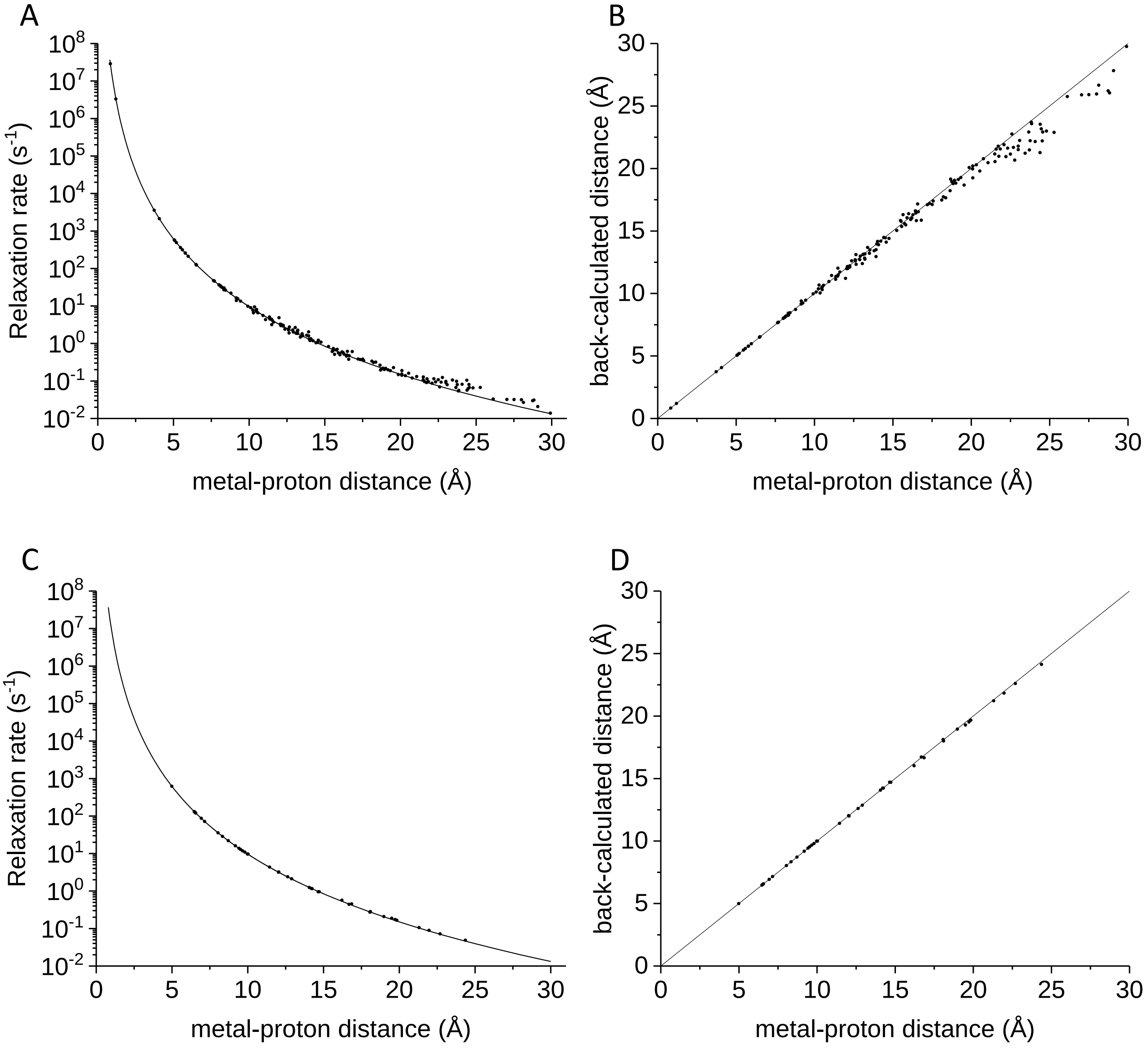

Figure S3. (A) Paramagnetic relaxation rates calculated at 500 MHz for Cu^{2+}-plastocyanin exchangeable (amide and hydroxyl) protons, in perdeuterated conditions. The line indicates the rates predicted with the Solomon equation. (C) Paramagnetic relaxation rates for isoleucine, leucine and valine methyl protons, assuming perdeuteration of all other hydrogens. (B and D) Agreement between metal-proton distances as measured in the PDB 2GIM structure and back-calculated from the predicted R_1 shown in panels A and C, respectively.

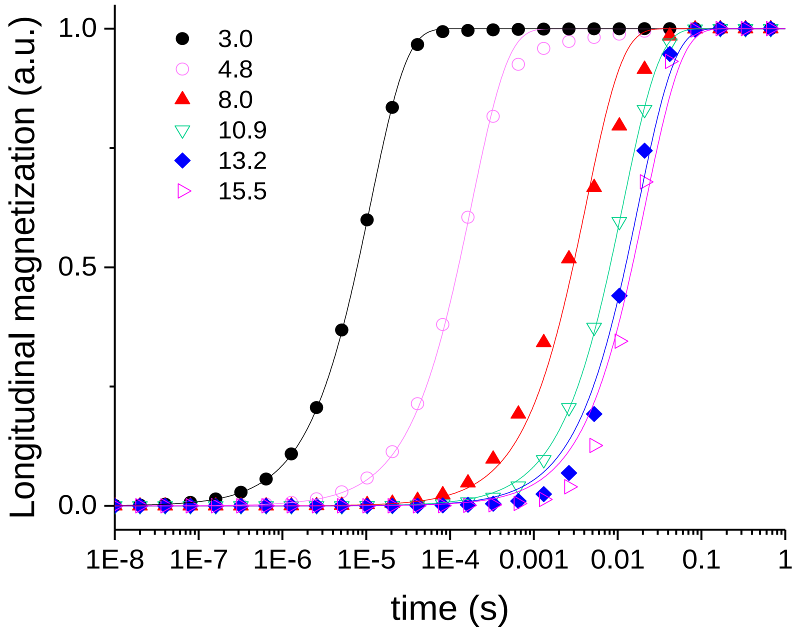

Figure S4. Magnetization recovery after a 90° pulse for protons at 3.0, 4.8, 8.0, 10.9, 13.2 and 15.5 Å from a Gd^{3+} ion, with electron relaxation time of 36 ns, in a macromolecule with a reorientation time of 500 ns, at 3 T. The lines indicate the monoexponential fit of the data.

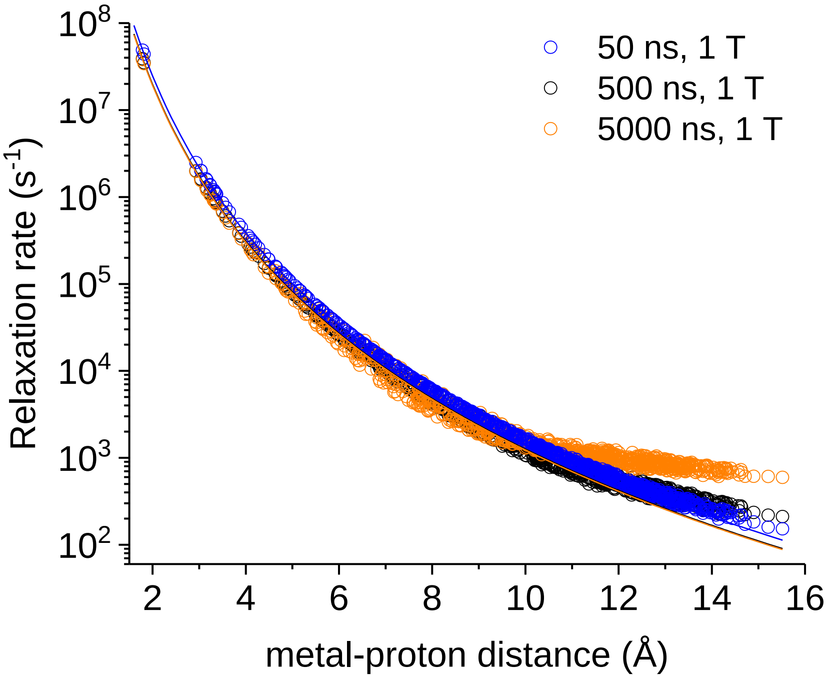

Figure S5. Relaxation rates calculated at 1 T for protons at different distance from a Gd^{3+} ion with electron relaxation time of 17 ns in the macromolecular model with reorientation time of 50, 500 or 5000 ns. The lines indicate the Solomon relaxation rates calculated for the same reorientation times (colored accordingly). The relaxation rate is calculated assuming Δ_t =0.015 cm^{-1} and τ_V= 20 ps, instead of Δ_t =0.030 cm^{-1} and τ_V= 20 ps, that provide 4.2 ns at 1 T.

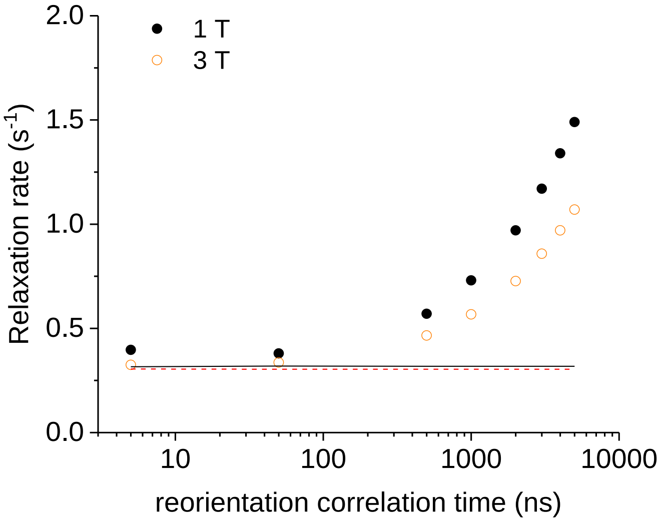

Figure S6. Bulk water proton relaxation rates calculated at 1 and 3 T as a function of the reorientation time of the macromolecular model (at 0.001 mol dm^{-3} concentration) with a $S = 1/2$ ion and electron relaxation time of 4 ns, with 100 surface protons with exchange rate of 0.1 ms. The bulk water proton relaxation rates calculated with the Solomon equation at 1 and 3 T are shown as solid and dashed lines, respectively. In all calculations, an intrinsic diamagnetic rate of 0.3 s^{-1} is assumed.

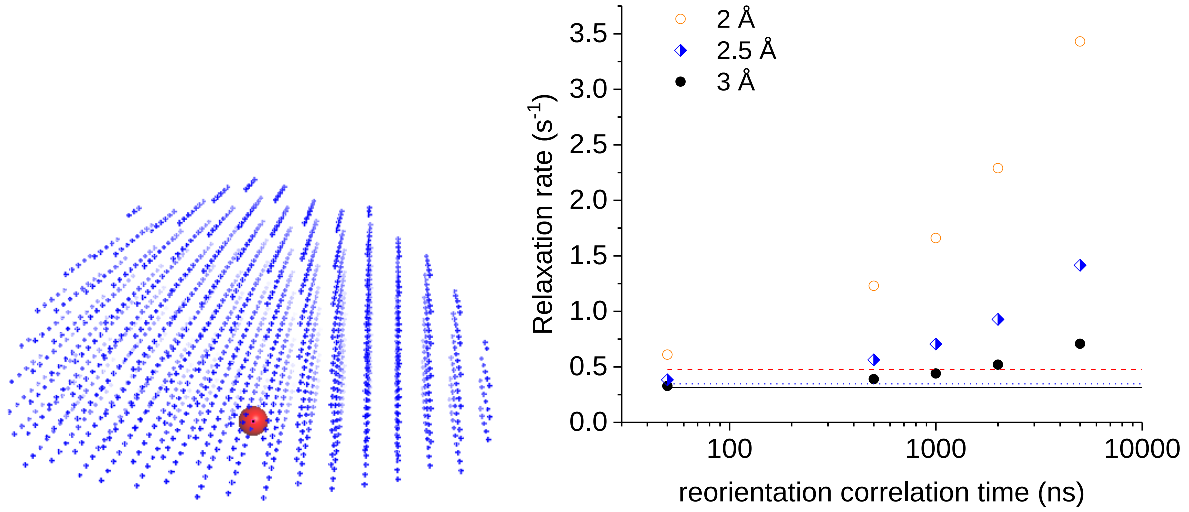

Figure S7. (A) Half-spherical structural model with protons in octahedral geometry and the metal ion (red sphere) in the center. (B) Bulk water proton relaxation rates at 1 T as a function of the reorientation time of a macromolecular sphere containing a Gd^{3+}-ion in the center and protons at distances of 2, 2.5 or 3 Å (see panel A), with 100 surface protons with exchange rate of 0.1 ms. The bulk water proton relaxation rates calculated with the Solomon equation are shown as lines. In all calculations, an intrinsic diamagnetic rate of 0.3 s^{-1} is assumed. The electron relaxation time of gadolinium is calculated assuming the typical values for the electron relaxation parameters, Δ_t =0.030 cm^{-1} and τ_V= 20 ps.
